# Supplementary material for: Comparative Analysis of the Genetic Diversity of Chilean Cultivated Potato Based on a Molecular Study of Authentic Herbarium Specimens and Present-Day Gene Bank Accessions
Source: Plants (Basel). 2022 Dec 31;12(1):174. doi: 10.3390/plants12010174 (PMC9823414; doi:10.3390/plants12010174)
Supplement: Supplementary file 1 [file plants-12-00174-s001.zip › FigureS1.pdf]

plants

MDPI

Article

Comparative Analysis of the Genetic Diversity of Chilean Cultivated Potato Based on a Molecular Study of Authentic Herbarium Specimens and Present-Day Gene Bank Accessions

Tatjana Gavrilenko*, Irena Chukhina, Olga Antonova, Ekaterina Krylova, Liliya Shipilina, Natalia Oskina and Ludmila Kostina

N.I. Vavilov All-Russian Institute of Plant Genetic Resources, Bolshaya Morskaya 42-44, 190000 Saint-Petersburg, Russia

*Correspondence: tatjana9972@yandex.ru

Supplementary Material

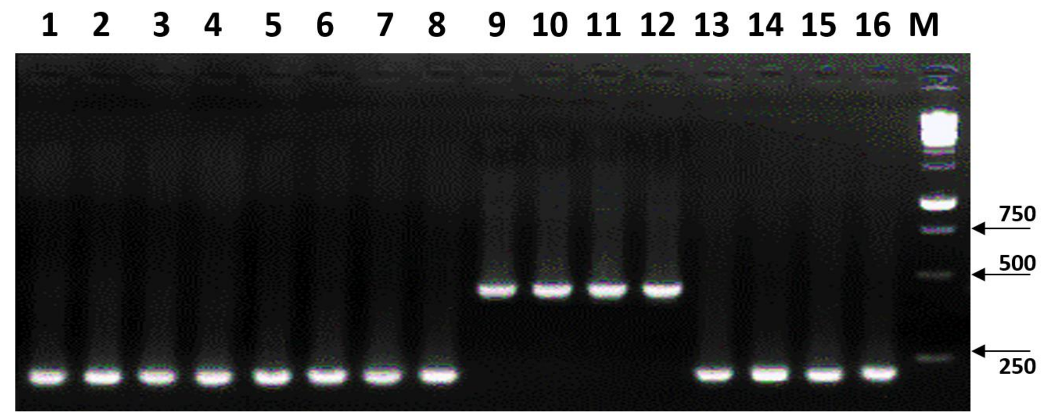

(a)

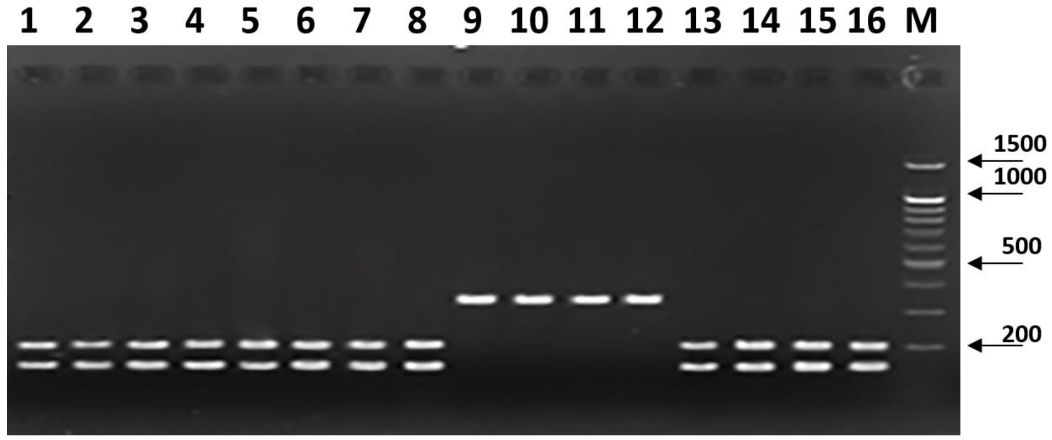

(b)

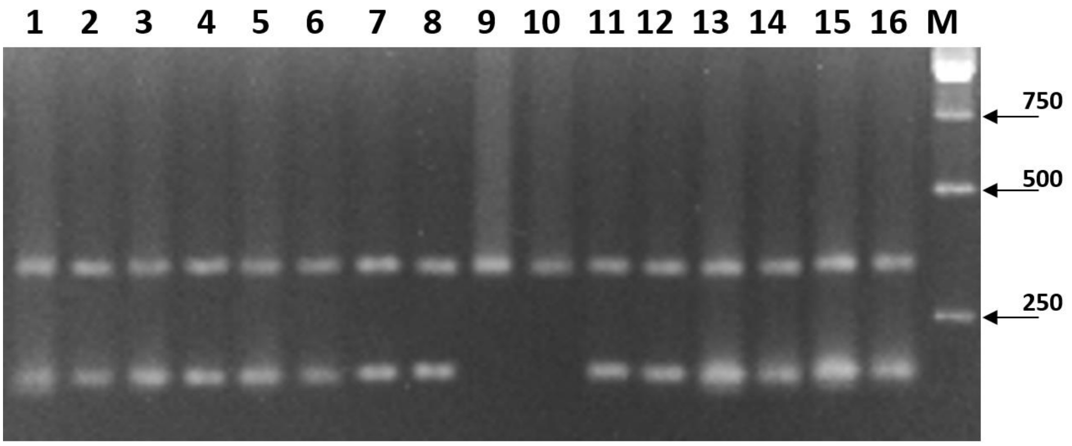
1 2 3 4 5 6 7 8 9 10 11 12 13 14 15 16 M
750
500
250

(c)

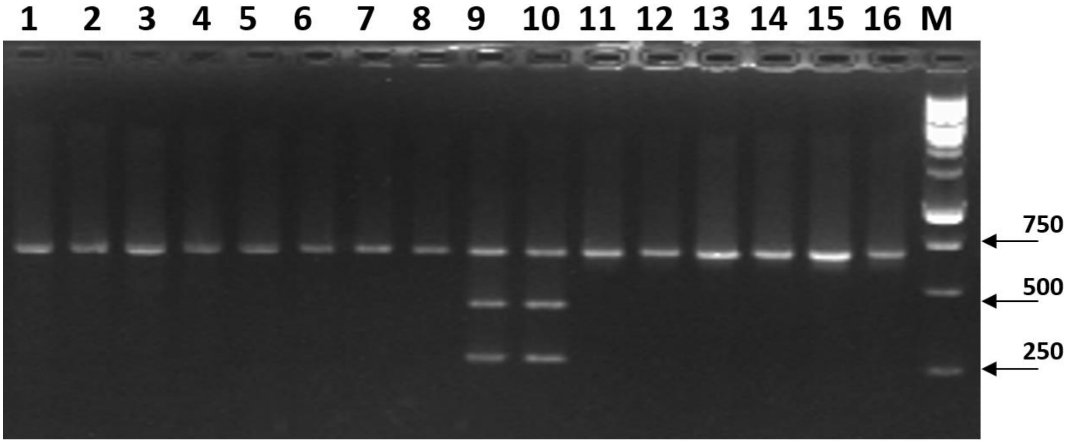
1 2 3 4 5 6 7 8 9 10 11 12 13 14 15 16 M
750
500
250

(d)

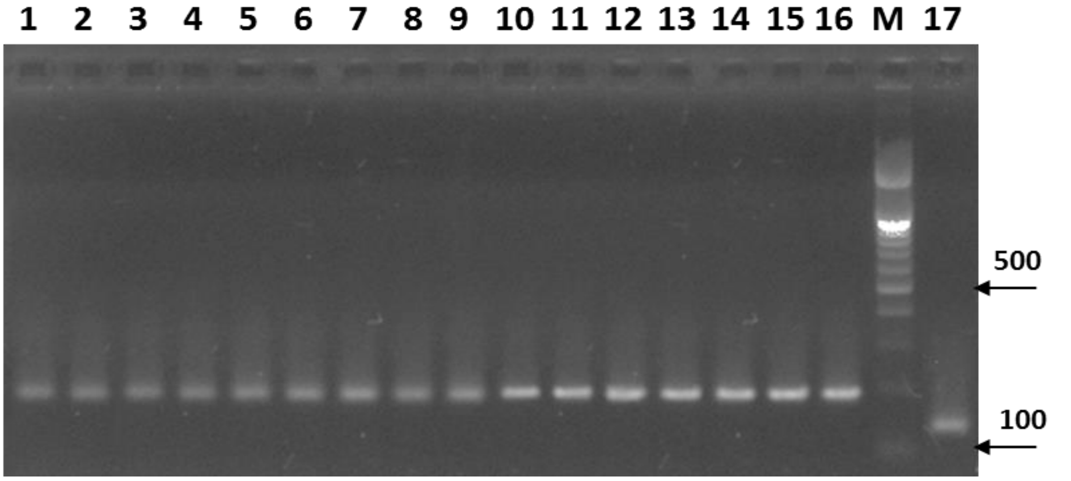
1 2 3 4 5 6 7 8 9 10 11 12 13 14 15 16 M 17
500
100

(e)

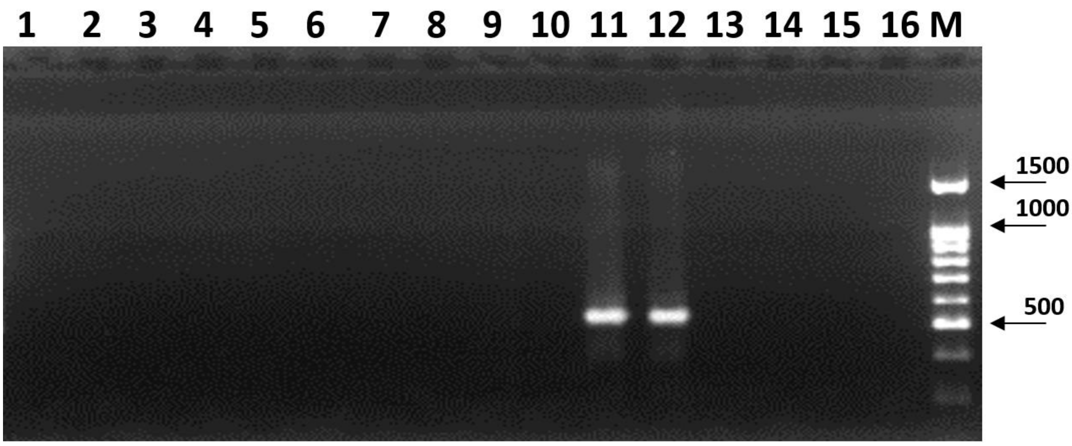

(f)

Figure S1. Detection of the cpDNA types (a-e) in Chilean specimens of *S. tuberosum* from the WIR herbaria (1 – 10) and in the living accessions from the VIR' field potato collection (11 - 16) in which cytoplasm type D was also determined (f). The following markers were used: (a) T marker (H1); (b) CAPS marker H2/HaeIII; (c) CAPS marker SAC/BamHI; (d) CAPS marker A22 (A/BamHI); (e) plastid SSR marker S (NTCP6); (f) the D (Region 1) marker. M– Molecular weight standards.

Herbarium specimen numbers: 1) N 1824, T-type cpDNA; 2) N 1939, T-type cpDNA; 3) N 2010, T-type cpDNA; 4) N 2040, T-type cpDNA; 5) N 2034, T-type cpDNA; 6) N 2025, T-type cpDNA; 7) N 2021, T-type cpDNA; 8) N 2014, T-type cpDNA; 9) N 2002, A-type cpDNA; 10) N 1940, A-type cpDNA.

Living accession: 11) k-3414, W-type cpDNA, cytoplasm type D; 12) k-5273, W-type cpDNA, cytoplasm type D; 13) k-3446, T-type cpDNA; 14) k-3475, T-type cpDNA; 15) k-3488, T-type cpDNA; 16) k-6092, T-type cpDNA; 17) *S. phureja*, k- 1817 (the control of S type of cpDNA).
