# Supplementary material for: Comparative Analysis of the Genetic Diversity of Chilean Cultivated Potato Based on a Molecular Study of Authentic Herbarium Specimens and Present-Day Gene Bank Accessions
Source: Plants (Basel). 2022 Dec 31;12(1):174. doi: 10.3390/plants12010174 (PMC9823414; doi:10.3390/plants12010174)
Supplement: Supplementary file 1 [file plants-12-00174-s001.zip › FigureS2.pdf]

plants

MDPI

Article

Comparative Analysis of the Genetic Diversity of Chilean Cultivated Potato Based on a Molecular Study of Authentic Herbarium Specimens and Present-Day Gene Bank Accessions

Tatjana Gavrilenko*, Irena Chukhina, Olga Antonova, Ekaterina Krylova, Liliya Shipilina, Natalia Oskina and Ludmila Kostina

N.I. Vavilov All-Russian Institute of Plant Genetic Resources, Bolshaya Morskaya 42-44, 190000 Saint-Petersburg, Russia
*Correspondence: tatjana9972@yandex.ru

Supplementary Material

aacttttgaactctattccttaattgagtatagaagtatagaacggtttagttacaagagttgaattcg
aggaaagtataaaatataggaaagtcccaggttaaataaaaaaaactaagactctaaactcaaatcaaaa
ataatgaaccttcaacctcaaattcctatttgaacaacttttattgttattgatccatttgaatcatta
ctaaactaaaatagcttactcaatctcgacgattgcttattcataggctattatgagttcaagacaagcc
gctatggtgaaattggtagacacgctgctcttaggaagcagtgctaatgcatctcggttcgagtccgagt
ggcggcatagcatcttctaaaaaggataaatagatcttataatgaattcaattcccgatttccattttag
aattatgtaattaagggactcttctttttaagatttttatgatattttcaaccttagagcatatatta
actcacatttcctttcgatcgtttcaattgtaattacaattcatttgataaccttttagtcgatgaaa
ttgtaaaactatacgattcgtcagaaaagggcataatagttactttttctgtataacaggattattagt
tactcgttggatttcttcaggacatttcccactaagcgatttatatgaatcattaattttcctttcatgg
agtttctcccttattcatataattccatatttcaaaaaaaatgttttaattttaagtaaaataactggac
caagtgctattttgacccaaggctttgctacttcaggtattttaactgaaatacaccaatctggaatatt
agtacctgctcttcaatctgagtggttaataatgcacgtaagtatgatgatattgggctatgcagccctt
ttatgt***ggatcg***ttattatcagtagcacttctagtgattacatttcgaaaaaacagaaagctttttat
aagagcaatggtttttaaacgagtcatttttcttgggtgaaaatgttgtcgaaaatacttcgtttttt
gtgctaaaaattattacaggtcccaattgattcaacaattggattattggagttatcgggttattagttt
aggatttactttttaaccataggaatcctttcgggagcggtatgggctaatgaagcgt

Figure S2. The fragment of cpDNA sequence DQ231562.1 (cv. Desiree, T-type) flanked by A-primers recommended by Hosaka and Sanetomo and positions of the A22 primer designed by us. The sequences of the A-primers [29] are underlined; the sequences of A22-primers are framed. The site *GGATCG* is marked in bold italic in which the base change to *GGATCC* forms an extra BamHI recognition site specific for A-type of cpDNA.
