# Supplementary material for: Comparative Analysis of the Genetic Diversity of Chilean Cultivated Potato Based on a Molecular Study of Authentic Herbarium Specimens and Present-Day Gene Bank Accessions
Source: Plants (Basel). 2022 Dec 31;12(1):174. doi: 10.3390/plants12010174 (PMC9823414; doi:10.3390/plants12010174)
Supplement: Supplementary file 1 [file plants-12-00174-s001.zip › TableS1.pdf]

plants

MDPI

Article

Comparative Analysis of the Genetic Diversity of Chilean Cultivated Potato Based on a Molecular Study of Authentic Herbarium Specimens and Present-Day Gene Bank Accessions

Tatjana Gavrilenko *, Irena Chukhina, Olga Antonova, Ekaterina Krylova, Liliya Shipilina, Natalia Oskina and Ludmila Kostina

N.I. Vavilov All-Russian Institute of Plant Genetic Resources, Bolshaya Morskaya 42-44, 190000 Saint-Petersburg, Russia
*Correspondence: tatjana9972@yandex.ru

Supplementary Material

Table S1. The herbarium specimens from the WIR and LE herbaria with their assigned haplotypes

№	Specimens number	The taxon name according to Bukasov (1933), or Juzepczuk (1937), or Lekhnovich (1978)	Herbarium label text 1	Indication on type herbarium specimen	cpDNA type	cpSSR haplotype	Chlorotype
**Chilean cultivated potato**							
1	1665	*S. tuberosum* var. *crassipedicellatum* Buk. et Lechn.	Chile, [Chiloe], Yutuy, Expedition of S. Juzepczuk. Reproduction: experimental base of VIR “Krasnyj Pakhar” near Leningrad, 1936, N 1970, к-1665, WIR-46295	core fund WIR	T	III	cpT_III
2	1824	*S. tuberosum* L.	[Chile], Santjago, [local name] "Papa amarilla". Reproduced near Leningrad, 1929, S. Juzepczuk, N 1824, field number № 4701, WIR-0071205	core fund WIR	T	III	cpT_III
3	1840b	*S. tuberosum* L. [2]	[Chile], Santjago, [local name] “Papa amarilla”. Reproduced near Leningrad, 1929, S. Juzepczuk, S. Bukasov, N 1840 в, field number № 4704, WIR-81686	core fund WIR	T	III	cpT_III
4	1923	*S. tuberosum* L.	Chile, Puerto Montt, [local name] "Papa coraila". Reproduced near Leningrad, 1929, S. Juzepczuk, N 1923, field number № 4716, WIR-0071268	core fund WIR	T	III	cpT_III
5	1930	*S. tuberosum* f. *monttiticum* Buk. et Lechn. [2]	[Chile], Chiloe, Chiloe, Puerto Montt, [local name] “Papa Rosada importada”; Reproduced near Leningrad, 1929, S. Juzepczuk, S. Bukasov, N 1930, field number № 4724, WIR- 81689	lectotypus WIR (Ovchinnikova et al., 2011)	T	III	cpT_III
6	1932a	*S. tuberosum* L.	Chile, Puerto Montt, [local name] "Papa lisa", Reproduced near Leningrad, 1929, S. Juzepczuk, N 1932a, field number № 4715, WIR-0071288	core fund WIR	T	III	cpT_III
7	1938	*S. tuberosum* var. *chilotanum* Buk. et Lechn.	[Chile], Chiloe, Ancud, [local name] "Papa americana". Reproduced near Leningrad, 1929, S. Juzepczuk, N 1938, field number № 4731, WIR-0071213	lectotypus WIR (Ovchinnikova et al., 2012)	T	III	cpT_III

8	1939	*S. tuberosum* var. *chilotanum* Buk. et Lechn.	Chile, [Chiloe], Ancud, [local name] "Papa American[a]". Reproduced near Leningrad, 1929, S. Juzepczuk, N 1939, field number № 4732, WIR-0071226	syntypus WIR	T	III	cpT_III
9	1940	*S. tuberosum* var. *multibaccatum* Buk. et Lechn. [2]	[Chile], Chiloe, Ancud, [local name] "Papa bastonesa". Reproduced near Leningrad, 1929, S. Juzepczuk, S. Bukasov, N 1940, field number № 4734, WIR- 81692	lectotypus WIR (Ovchinnikova et al. 2011)	A	II	cpA_II
10	1944	*S. tuberosum* f. *chaped* Buk. et Lechn.	[Chile], Chiloe, Ancud, [local name] "Papa Chaped". Reproduced near Leningrad, 1929, S. Juzepczuk, N 1944, field number № 4737, WIR-81695	lectotypus WIR (Ovchinnikova et al. 2011)	T	III	cpT_III
11	1955a	*S. tuberosum* L.	Chile, [Chiloe], Ancud, [local name] "Papa American[a]". Reproduced near Leningrad, 1929, S. Juzepczuk, N 1955a, field number № 4739, WIR-0071220	core fund WIR	T	III	cpT_III
12	1958	*S. tuberosum* L.	Chile, [Chiloe], Castro, [local name] "Papa largucha". Reproduced near Leningrad, 1929, S. Juzepczuk, N 1958, field number № 4745, WIR-0071281	core fund WIR	T	III	cpT_III
13	1959	*S. tuberosum* L.	[Chile], Chiloe, Castro, [local name] "Papa bastonesa". Reproduced near Leningrad, 1929, S. Juzepczuk, N 1959, field number № 4746, WIR-81698	core fund WIR	T	III	cpT_III
14	1962	*S. tuberosum* f. *viride* Buk. et Lechn.	[Chile], Chiloe, Yutuy. [Reproduced near Leningrad], 1929, S. Juzepczuk, S. Bukasov, N 1962, field number № 4752, WIR- 81702	lectotypus WIR (Ovchinnikova et al. 2012)	T	III	cpT_III
15	1970	*S. tuberosum* var. *crassipedicellatum* Buk. et Lechn. [2]	[Chile], Chiloe, Yutuy, [local name] "California". Reproduced near Leningrad, 1929, S. Juzepczuk, S. Bukasov, N 1970, field number № 4760, WIR-81705	lectotypus WIR (Chukhina et al. 2016)	T	III	cpT_III
16	1971	*S. tuberosum* var. *rubrisuturatum* Buk. et Lechn.	Chile, Chiloe, Yutuy, farmyard, [local name] "Coleo", 1929, S. Juzepczuk, det. V. Lechnovich, N 1971, WIR-81683	lectotypus WIR (Ovchinnikova et al. 2011)	T	III	cpT_III
17	1975	*S. tuberosum* f. *araucanum* Buk. et Lechn. [2]	[Chile], Chiloe, Yutuy, [local name] "Araucana"; Reproduced near Leningrad, 1929, S. Juzepczuk, S. Bukasov, N 1975, field number № 4675, WIR-82779	lectotypus WIR (Ovchinnikova et al. 2011)	T	III	cpT_III
18	1979a	*S. tuberosum* L.	Chile, [Chiloe], Yutuy, [local name] "Papa maude". Reproduced near Leningrad, 1929, S. Juzepczuk, N 1979a, field number № 4770, WIR-0071301	core fund WIR	T	III	cpT_III
19	1979b	*S. tuberosum* L.	[Chile], Chiloe, Yutuy, [local name] "Papa maude". Reproduced near Leningrad, 1929, S. Juzepczuk, N 1979b, field number № 4771/1979II, WIR-82783	core fund WIR	T	III	cpT_III
20	1980	*S. tuberosum* f. *crassifilamentum* Buk. et Lechn.	[Chile], Chiloe, Yutuy, [local name] "Reina Imperada". Reproduced near Leningrad, 1931, S. Juzepczuk, S. Bukasov, N 1980, WIR-82787	lectotypus WIR (Ovchinnikova et al. 2011)	T	III	cpT_III
21	1982a	*S. tuberosum* L.	[Chile], Chiloe, Jutuy [Yutuy], [local name] "Papa seda". Reproduced near Leningrad, 1931, S. Juzepczuk», N 1982a, WIR-82791	core fund WIR	T	III	cpT_III
22	1986	*S. tuberosum* f. *pillicuma* Buk. et Lechn. [2]	[Chile], Chiloe, Iutuy [Yutuy], [local name] "Papa pillicuma". Reproduced near Leningrad, 1929, S. Juzepczuk, S. Bukasov, N 1986, field number № 4778, WIR-82791	lectotypus WIR (Ovchinnikova et al. 2011)	T	III	cpT_III

23	1987a	*S. tuberosum* L.	[Chile], Chiloe, Iutuy [Yutuy], [local name] "Huinco". Reproduced near Leningrad, 1929, S. Juzepczuk, N 1987a, field number № 4779, WIR-82861	core fund WIR	T	III	cpT_III
24	1987b	*S. tuberosum* L.	[Chile], Chiloe, Iutuy [Yutuy], [local name] "Huinco". Reproduced near Leningrad, 1929, S. Juzepczuk, N 1987b, field number № 4780a, WIR-82866	core fund WIR	T	III	cpT_III
25	1990	*S. tuberosum* L.	Chile, [Chiloe], Yutuy, [local name] "Papa murta". Reproduced near Leningrad, 1929, S. Juzepczuk, N 1990, field number № 4785, WIR-0071310	core fund WIR	T	III	cpT_III
26	1991	*S. tuberosum* f. *obliquum* Buk. et Lechn. [2]	[Chile], Chiloe, Iutuy [Yutuy], [local name] "Papa Mantequilla". Reproduced near Leningrad, 1929, S. Juzepczuk, S. Bukasov, N 1991, field number, № 4786, WIR-0071294	lectotypus WIR (Ovchinnikova et al. 2011)	T	III	cpT_III
27	1993	*S. tuberosum* var. *ele-gans* Buk. et Lechn. [2]	Chile, [Chiloe], Yutuy, [local name] "Papa Guapa". Reproduced near Leningrad, 1929, S. Juzepczuk, S. Bukasov, N 1993, field number № 4788, WIR-0071275	isolectotypus WIR (Chukhina et al. 2016)	T	III	cpT_III
28	1994	*S. tuberosum* L. f. y*utuense* Buk. et Lechn. [2]	[Chile], Chiloe, Yutuy, [local name] "Francesa negra". Reproduced near Leningrad, 1929, S. Juzepczuk, S. Bukasov, N 1994, field number № 4789, WIR-82882	isolectotypus WIR (Chukhina et al. 2016)	T	III	cpT_III
29	1996	*S. tuberosum* L. f. *pichuna* Buk. et Lechn.	Chile, Chiloe, Yutuy, [local name] "Pichuna", 1930, S. Juzepczuk, det. V. Lechnovich, N 1996, WIR-95815	lectotypus WIR (Ovchinnikova et al. 2011)	T	III	cpT_III
30	1999	*S. tuberosum* L.[2]	[Chile], Chiloe, Iutuy [Yutuy], Aguatao, [local name] "Papa cauchao". Reproduced near Lenin-grad, 1929, S. Juzepczuk, N 1999, field number № 4801, WIR-95849	core fund WIR	T	III	cpT_III
31	2000b II	*S. tuberosum* L.	[Chile], Chiloe, Iutuy [Yutuy], Agua tao, [local name] "Papa caballera". Reproduced near Lenin-grad, 1929, S. Juzepczuk, N 2000вII, field number № 4794, WIR-95923	core fund WIR	T	III	cpT_III
32	2001	*S. tuberosum* L.	Chile, [Chiloe], Jutuy [Yutuy], Agua tao, [local name] "Papa cobra". Reproduced near Leningrad, 1929, S. Juzepczuk, N 2001, field number № 4796, WIR-0071262	core fund WIR	T	III	cpT_III
33	2002	*S. tuberosum* L.	Chile, [Chiloe], Jutuy [Yutuy], Aguatao, [local name] "Papa Bolera". Reproduced near Leningrad, 1929, S. Juzepczuk, N 2002, field number № 4797, WIR-0071250	core fund WIR	A	II	cpA_II
34	2004	*S. tuberosum* L.	Chile, [Chiloe], Jutuy [Yutuy], Aguatao, [local name] "Papa bline". Reproduced near Leningrad, 1929, S. Juzepczuk , N 2004, field number № 4799, WIR-0071239	core fund WIR	T	III	cpT_III
35	2005	*S. tuberosum* var. *recurvatum* Buk. et Lechn. [2]	Chile, [Chiloe], Jutuy [Yutuy], Aguatao, [local name] "Papa pachacona". Reproduced near Lenin-grad, 1929, S. Juzepczuk, N 2005, field number № 4800, WIR-0071317	lectotypus WIR (Ovchinnikova et al. 2011)	T	III	cpT_III
36	2006	*S. tuberosum* f. *latum* Buk. et Lechn.	[Chile], Chiloe, Yutuy, Aguatao, [local name] "Papa Tempranera". Reproduced near Leningrad, 1929, S. Juzepczuk, S. Bukasov, N 2006, field num-ber № 4792, WIR-95924	lectotypus WIR (Ovchinnikova et al. 2011)	T	III	cpT_III

37	2007	*S. tuberosum* L.	Chile, [Chiloe], Jutuy [Yutuy], Aguatao, [local name] "Papa cauchao". Reproduced near Leningrad, 1929, S. Juzepczuk, N 2007, field number № 4802, WIR-0071256	core fund WIR	T	III	cpT_III
38	2010	*S. tuberosum* var. *brevipilosum* Buk. et Lechn. [2]	[Chile], Chiloe, Castro, [local name] "Caballera". Reproduced near Leningrad, 1929, S. Juzepczuk, S. Bukasov, N 2010, field number № 4749, WIR-95925	lectotypus WIR (Ovchinnikova et al. 2011)	T	III	cpT_III
39	2020	*S. tuberosum* f. *acuminatum* Buk. et Lechn. [2]	[Chile], Chiloe, Meghaico [Mechaico]. [local name] "Papa bastonesa". Reproduced near Leningrad, 1929, S. Juzepczuk, S. Bukasov, N 2020, field number № 4806, WIR 97456	lectotypus WIR (Ovchinnikova et al. 2011)	T	III	cpT_III
40	2021	*S. tuberosum* var. *villaroella* Buk. et Lechn.	Chile, [Chiloe], Meghaico [Mechaico], [local name] "Papa Vilaroela ["Villaroela"]. Reproduced near Leningrad, 1929, S. Juzepczuk, S. Bukasov, N 2021, field number № 4807, WIR-0071330	lectotypus WIR (Ovchinnikova et al. 2011)	T	III	cpT_III
41	2022	*S. tuberosum* f. *infectum* Buk. et Lechn.	[Chile], Chiloe, Mechaico, [local name] "Bolera", 1929, S. Juzepczuk, det. V. Lechnovich, N 2022, WIR-0071245	lectotypus WIR (Ovchinnikova et al. 2011)	T	III	cpT_III
42	2025a	*S. tuberosum* f. *thalassinum* Buk. et Lechn.	Chile, [Chiloe], Quetalmahue, [local name] "Papa azulera". Reproduced near Leningrad, 1929, S. Juzepczuk, N 2025a, field number № 4811, WIR-0071232	lectotypus WIR	T	III	cpT_III
43	2028	*S. tuberosum* L. [2]	[Chile], Chiloe, Quetalmahue, [local name] "Papa nalca". Reproduced near Leningrad, 1929, S. Juzepczuk, N 2028, field number № 4814, WIR-97457	core fund WIR	T	III	cpT_III
44	2029	*S. tuberosum* L.	[Chile], Chiloe, Quetalmahue, [local name] "Cobra". 1931, S. Juzepczuk, N 2029, WIR-97458	core fund WIR	T	III	cpT_III
45	2030c	*S. tuberosum* L. [2]	[Chile], Chiloe, Quetalmahue, [local name] "Mahuihue". Reproduced near Leningrad, 1929, S. Juzepczuk, N 2030c, field number № 4816, WIR-97459	core fund WIR	T	III	cpT_III
46	2034	*S. tuberosum* f. *sebastianum* Buk. et Lechn.	Chile, island San Sebastiano [Isla Dona Sebastiana?], [local name] "Soldada", 1929, S. Juzepczuk, det. V. Lechnovich, N 2034, WIR-97460	lectotypus WIR (Ovchinnikova et al. 2011)	T	III	cpT_III
47	2035	*S. tuberosum* L. [2]	[Chile], Chiloe, island S[an] Sebastiano [Isla Dona Sebastiana?], [local name] "Araucana blanca". Reproduced near Leningrad, 1929, S. Juzepczuk, N 2035, field number № 4820, WIR-97461	core fund WIR	T	III	cpT_III
48	2038	*S. tuberosum* f. *mahuinhue* Buk. et Lechn. [2]	[Chile], Chiloe, island S[an] Sebastiano [Isla Dona Sebastiana?], [local name] "Montequilla rosada"; Reproduced near Leningrad, 1929, S. Juzepczuk, S. Bukasov, N 2038, field number № 4823, WIR-97975	lectotypus WIR (Ovchinnikova et al. 2011)	T	III	cpT_III
49	2040	*S. tuberosum* L. f. *contortum* Buk. et Lechn. [2]	[Chile], Chiloe, island S[an] Sebastiano [Isla Dona Sebastiana?], [local name "Australia"]. Reproduced near Leningrad, 1929, det. S. Juzepczuk, S. Bukasov, N 2040, field number № 4825a, WIR-97978	lectotypus WIR (Ovchinnikova et al. 2012)	T	III	cpT_III
**Wild Chilean species according to Russian taxonomists**							

50	072	*S. leptostigma* Juz. [3]	Chile, Temuco, [local name] "Silvestre", № 072, S. Juzepzuk, WIR-42776	core fund WIR	T	III	cpT_III
51	2015	*S. leptostigma* Juz. [3]	Chile, [Chiloe], Cucao, [local name] "Silvestre", Juzepczuk, N 2015, № 073, det. S. Bukasov, WIR-0098243	neotypus WIR (Chukhina et al. 2017)	T	III	cpT_III
52	1831	*S. maglia* Schlechtd.	1831, LE	core fund LE	–	II	–
53	2871	*S. maglia* Schlechtd.	Chile, Pacific Chilean Group. N 2871. Herbarium is collected at the VIR experimental station in the town of Pushkin, D-35, 24 VIII 1954, leg. Korenovkina. WIR-77604	core fund WIR	A	II	cpA_II
54	2014	*S. molinae* Juz. [3]	Chile, Chiloe, Expedition of S. Juzepczuk (1927) [1928]. Reproduction: VIR experimental base "Krasnyj Pakhar'" near Leningrad, 23 VIII 1931, leg. V. Lechnovich, N 2014, field number № 078, WIR-97979	syntypus WIR (Chukhina et al. 2017)	T	III	cpT_III
55	2059	*S. molinae* Juz. [3]	Chile, Chiloe, Cucao, Expedition of S. Juzepchuk (1927) [1928]. Reproduction: experimental base of VIR "Krasnyj Pakhar" near Leningrad, 29 VIII 1931, leg. V. Lechnovich, det. S. Bukasov, N 2059, field number № 078, WIR-50098	syntypus WIR (Chukhina et al. 2017)	T	III	cpT_III
56	2902	*S. molinae* Juz. [3]	Chile, Pacific Chilean Group. N 2902. Herbarium is collected at the VIR experimental station in the town of Pushkin, 24 VIII 1954, leg. Korenovkina, WIR-0077722	core fund WIR	T	III	cpT_III
57	87	*S. molinae* Juz. [3]	South America. Reproduction: VIR Pushkin's laboratory, D-230, № 87, 1 VIII 1963, leg. Kalinina, det. Roborovskaya. WIR-0084148	core fund WIR	T	III	cpT_III
58	11289	*S. ochoanum* Lechn.	Chile, Insula Guaiteca [Isla Gran Guaiteca]. Leg. Carlos Ochoa. Reproduced at the VIR experimental station in the town of Pushkin, 25 VIII 1978, leg. Turuleva L.M., det. Dr. Vadim S. Lechnovitch, k-11289, WIR-98143	syntypus WIR (Chukhina et al. 2017)	T	III	cpT_III
59	11290	*S. ochoanum* Lechn.	Chile, Insula Guaiteca [Isla Gran Guaiteca]. Leg. Carlos Ochoa. Reproduction at the VIR Experimental Station in the town of Pushkin, 25 VIII 1978, leg. Turuleva L. M., det. Dr. Vadim S. Lechnovitch, k-11290, WIR-98326	syntypus WIR (Chukhina et al. 2017)	T	III	cpT_III
60	11288	*S. zykinii* Lechn.	[Chile, Chiloe], Ancud, Ochoa, sepulereto. Reproduced near Leningrad, 08 VIII 1978, leg. A. A. Davydova, k-11288, WIR-41785	syntypus WIR (Chukhina et al. 2017)	T	III	cpT_III
61	7600	*S. zykinii* Lechn.	South America, in statu silvestri insula Chiloe, 43° oceani litorale. Collected by A.G. Zykin on Chiloe Island in the wild state. Reproduced at the Pavlovsk Experimental Station. 27 VIII 1968, leg. Sharapova, k-7600, WIR-25830	syntypus WIR (Chukhina et al. 2017)	T	III	cpT_III

[1] Clarifications in square brackets made by the authors of present paper based on the publication of Bukasov (1933).

[2] These 18 specimens, when studied by monographs, were originally defined as S. tuberosum s. str. (Bukasov, 1933), but, unfortunately, when making a herbarium list in 1929 for these specimens, herbarium labels with a printed name «*Solanum andigenum*" were used.

[3] Ovchinnikova et al. (2011) indicated that the names of *S. molinae* Juz. and *S. leptostigma* Juz. are «…not validly published». However, the names *S. leptostigma* and *S. molinae* were validly published by Juzepczuk in 1937 (Juzepczuk 1937). Lectotype of *S. molinae* (Chukhina et al. 2017) is preserved in the Herbarium LE, syntypes of *S. molinae* and neotype of *S. leptostigma* are stored in the Herbarium WIR (Chukhina et al. 2016).
