# Supplementary material for: Comparative Analysis of the Genetic Diversity of Chilean Cultivated Potato Based on a Molecular Study of Authentic Herbarium Specimens and Present-Day Gene Bank Accessions
Source: Plants (Basel). 2022 Dec 31;12(1):174. doi: 10.3390/plants12010174 (PMC9823414; doi:10.3390/plants12010174)
Supplement: Supplementary file 1 [file plants-12-00174-s001.zip › TableS2.pdf]

plants

MDPI

Article

Comparative Analysis of the Genetic Diversity of Chilean Cultivated Potato Based on a Molecular Study of Authentic Herbarium Specimens and Present-Day Gene Bank Accessions

Tatjana Gavrilenko*, Irena Chukhina, Olga Antonova, Ekaterina Krylova, Liliya Shipilina, Natalia Oskina and Ludmila Kostina

N.I. Vavilov All-Russian Institute of Plant Genetic Resources, Bolshaya Morskaya 42-44, 190000 Saint-Petersburg, Russia
*Correspondence: tatjana9972@yandex.ru

Supplementary Material

Table S2a. Allelic composition of four haplotypes (each including more than one accession per haplotype) at 15 polymorphic plastid SSR loci

**cpSSR haplotype**		**#II**	**#III**	**#V**	**#Chl 3414**	
No. of herbarium specimens		4	57	0	0	
No. of living accessions		2	39	4	1	
#	**Loci:**					
1	STCP1	120	117	119	119	
2	STCP2	124	125	123	123	
3	STCP3	88	87	88	87	
4	STCP4	176	171	171	171	
5	STCP5	171	170	170	170	
6	STCP6	95	96	97	98	
7	STCP7	126	126	127	127	
8	STCP9	188	188	188	188	No polymorphism
9	STCP10	176	177	176	176	
10	STCP11	136	136	136	136	No polymorphism
11	STCP12	192	192	192	192	No polymorphism
12	STCP13	128	128	128	128	No polymorphism
13	NTCP6	174	173	174	174	
14	NTCP12	127	125	125	125	
15	NTCP14	151	149	151	150	

Table S2b. Designation of chlorotypes, plastid DNA types, cpSSR haplotypes, mitotypes and cytoplasm types.

Chloroplast type, this study	cpDNA type according to Hosaka and Sanetomo, 2012 [29]	cpSSR haplotype according to Gavrilenko et al., 2013 [36]	mtDNA type according to Lossl et al., 2000 [30]	Cytoplasm types according to Hosaka, Sanetomo, 2012 [29]
cpA_II	A	#II	β	A
cpT_III	T	#III	β	T
cpW_ V	W	#V	α	D
cpW_ Chl 3414	W	#Chl 3414	α	D
