# Supplementary material for: Comparative Analysis of the Genetic Diversity of Chilean Cultivated Potato Based on a Molecular Study of Authentic Herbarium Specimens and Present-Day Gene Bank Accessions
Source: Plants (Basel). 2022 Dec 31;12(1):174. doi: 10.3390/plants12010174 (PMC9823414; doi:10.3390/plants12010174)
Supplement: Supplementary file 1 [file plants-12-00174-s001.zip › TableS3.pdf]

plants

MDPI

Article

Comparative Analysis of the Genetic Diversity of Chilean Cultivated Potato Based on a Molecular Study of Authentic Herbarium Specimens and Present-Day Gene Bank Accessions

Tatjana Gavrilenko*, Irena Chukhina, Olga Antonova, Ekaterina Krylova, Liliya Shipilina, Natalia Oskina and Ludmila Kostina

N.I. Vavilov All-Russian Institute of Plant Genetic Resources, Bolshaya Morskaya 42-44, 190000 Saint-Petersburg, Russia
* Correspondence: tatjana9972@yandex.ru

Supplementary Material

Table S3. The living Chilean accessions maintained in the VIR field potato collection which were used in this study with their assigned cpDNA-, mtDNA-types, cytoplasm types, cpSSR haplotypes, and data of molecular screening with DNA markers of the *R1* and *R3a* genes conferring race-specific resistance to late blight diseases.

№	VIR accession number	VIR expeditions, year	Donor genebank	Received by the VIR, year	cpDNA-type[1]	cpSSR-haplotype[2]	Chlorotype[3]	mtDNA-type[4]	Cytoplasm type[1]	*R1*	*R3a*
1	k-3385	Zhukovsky (VIR), 1958		1958	T	#III	cpT_III	β	T	0	0
2	k-3400	Zhukovsky (VIR), 1958		1958	T	#III	cpT_III	β	T	0	0
3	k-3407a	Zhukovsky (VIR), 1958		1958	T	#III	cpT_III	β	T	0	0
4	k-3414	Zhukovsky (VIR), 1958		1958	W	#Chl 3414	cpW_ Chl 3414	α	D	1	0
5	k-3446	Zhukovsky (VIR), 1958		1958	T	#III	cpT_III	β	T	0	0
6	k-3456	Zhukovsky (VIR), 1958		1958	T	#III	cpT_III	β	T	0	0
7	k-3471	Zhukovsky (VIR), 1958		1958	T	#III	cpT_III	β	T	0	0

8	k-3475	Zhukovsky (VIR), 1958		1958	T	#III	cpT_III	β	T	0	0
9	k-3484	Zhukovsky (VIR), 1958		1958	T	#III	cpT_III	β	T	0	0
10	k-3485	Zhukovsky (VIR), 1958		1958	T	#III	cpT_III	β	T	0	0
11	k-3488	Zhukovsky (VIR), 1958		1958	T	#III	cpT_III	β	T	0	0
12	k-5273		Gross-Lüsewitz, Germany	1964	W	#V	cpW_ V	α	D	0	1
13	k-6008		CPC	1964	T	#III	cpT_III	β	T	0	0
14	k-6032		CPC	1964	T	#III	cpT_III	β	T	0	0
15	k-6052		CPC	1964	T	#III	cpT_III	β	T	0	0
16	k-6079		CPC	1964	T	#III	cpT_III	β	T	0	0
17	k-6092		CPC	1964	T	#III	cpT_III	β	T	0	0
18	k-6093		CPC	1964	T	#III	cpT_III	β	T	0	0
19	k-6107		CPC	1964	T	#III	cpT_III	β	T	0	0
20	k-6111		CPC	1964	T	#III	cpT_III	β	T	0	0
21	k-6113		CPC	1964	T	#III	cpT_III	β	T	0	0
22	k-6116		CPC	1964	T	#III	cpT_III	β	T	0	0
23	k-7501	Zykin (VIR), 1967		1967	T	#III	cpT_III	β	T	0	0
24	k-7504	Zykin (VIR), 1967		1967	T	#III	cpT_III	β	T	0	0
25	k-7520	Zykin (VIR), 1967		1967	T	#III	cpT_III	β	T	0	0
26	k-7523	Zykin (VIR), 1967		1967	T	#III	cpT_III	β	T	0	0
27	k-7528	Zykin (VIR), 1967		1967	T	#III	cpT_III	β	T	0	0
28	k-7529	Zykin (VIR), 1967		1967	T	#III	cpT_III	β	T	0	0
29	k-7530	Zykin (VIR), 1967		1967	T	#III	cpT_III	β	T	0	0
30	k-7535	Zykin (VIR), 1967		1967	W	#V	cpW_ V	α	D	1	0
31	k-7539	Zykin (VIR), 1967		1967	A	#II	cpA_II	β	A	0	0
32	k-7540	Zykin (VIR), 1967		1967	T	#III	cpT_III	β	T	0	0

33	k-7543	Zykin (VIR), 1967		1967	T	#III	cpT_III	β	T	1	0
34	k-7550	Zykin (VIR), 1967		1967	T	#III	cpT_III	β	T	0	0
35	k-7568	Zykin (VIR), 1967		1967	W	#V	cpW_ V	α	D	1	0
36	k-7573	Zykin (VIR), 1967		1967	T	#III	cpT_III	β	T	0	0
37	k-7576a	Zykin (VIR), 1967		1967	A	#II	cpA_II	β	A	0	0
38	k-7579	Zykin (VIR), 1967		1967	T	#III	cpT_III	β	T	0	0
39	k-7580	Zykin (VIR), 1967		1967	T	#III	cpT_III	β	T	0	0
40	k-7583	Zykin (VIR), 1967		1967	T	#III	cpT_III	β	T	0	0
41	k-7586	Zykin (VIR), 1967		1967	W	#V	cpW_ V	α	D	1	0
42	k-7589	Zykin (VIR), 1967			T	#III	cpT_III	β	T	0	0
43	k-7599	Zykin (VIR), 1967		1967	T	#III	cpT_III	β	T	1	0
44	k-10648		Gross-Lüsewitz, Germany	1972	T	#III	cpT_III	β	T	0	0
45	k-23989		USA, NRSP-6	2009	T	#III	cpT_III	β	T	0	0
46	PI 245835		USA, NRSP-6	2009	T	#III	cpT_III	β	T	0	0

[1] plastid DNA (cpDNA-) types and cytoplasm types – designations according to Hosaka and Sanetomo 2012;
[2] cpSSR haplotypes – designations according to Gavrilenko et al. 2013;
[3] chlorotypes – present study;
[4] mitotypes (mtDNA-types) – designations according to Löss et al. 2000.
CPC - The Common-wealth Potato Collection of James Hutton Institute in Dundee, UK; NRSP-6 - United States Potato Genebank.
NRSP-6 – the US Potato Genebank.
