# Supplementary material for: Comparative Analysis of the Genetic Diversity of Chilean Cultivated Potato Based on a Molecular Study of Authentic Herbarium Specimens and Present-Day Gene Bank Accessions
Source: Plants (Basel). 2022 Dec 31;12(1):174. doi: 10.3390/plants12010174 (PMC9823414; doi:10.3390/plants12010174)
Supplement: Supplementary file 1 [file plants-12-00174-s001.zip › TableS4.pdf]

plants

MDPI

*Article*

# Comparative Analysis of the Genetic Diversity of Chilean Cultivated Potato Based on a Molecular Study of Authentic Herbarium Specimens and Present-Day Gene Bank Accessions

**Tatjana Gavrilenko*, Irena Chukhina, Olga Antonova, Ekaterina Krylova, Liliya Shipilina, Natalia Oskina and Ludmila Kostina**

N.I. Vavilov All-Russian Institute of Plant Genetic Resources, Bolshaya Morskaya 42-44, 190000 Saint-Petersburg, Russia
* Correspondence: tatjana9972@yandex.ru

## Supplementary Material

**Table S4 a-c.** Molecular markers, primers and their sequence, annealing temperature (T°m), restriction enzyme used to analyze DNA polymorphism in herbarium specimens and in living accessions of Chilean potatoes (primers that had reliable amplification in old herbarium specimens are indicated in bold type).

**Table S4a.** Plastid and mitochondrial DNA markers for detection of cpDNA-, mtDNA- and cytoplasm types in herbarium specimens and in living accessions of Chilean potatoes.

| Locus | DNA marker | Primers name | Restrictase | Primers sequences (5'→ 3') | T°m | References |
|---|---|---|---|---|---|---|
| **The PCR markers for detection of cpDNA types** | | | | | | |
| *ndhC/ trnV* | T | H1 | – | F: GGAGGGGTTTTTCTTGGTTG<br>R: AAGTTTACTCACGGCAATCG | 55 | [32] |
| *rps16/ trnQ* | S | NTCP6 | – | F: GGTTCGAATCCTTCCGTC<br>R: GATTCTTTCGCATCTCGATTC | 63→58 | [63] |
| *cemA* | SAC | SAC | BamHI | F: TTGGAGTTGTTGCGAATGAG<br>R: GTTCCCTAGCCACGATTCTG | 60 | [29] |
| *rpl32/ ccsA* | A | A | BamHI | F: AACTTTTTGAACTCTATTCCTTAATTG<br>R: ACGCTTCATTAGCCCATACC | 60 | [29] |
| *rpl32/ ccsA* | A22 | A22 | BamHI | F: TAACTCACATTTCCTTTTCG<br>R: ACGCTTCATTAGCCCATAC | 53→48 | This study |
| *rbcL* | H2 | H2 | HaeIII | F: GCATCGAGCGTGTTGTTGGA<br>R: AGTCCACCGCGAAGACATTC | 55 | [32] |
| **Markers for detection of mtDNA types** | | | | | | |
| *rps10* | ALM4/5 | ALM_4<br>ALM_5 | – | F: AATAATCTTCCAAGCGGAGAG<br>R: AAGACTCGTGATTCAGGCAAT | 55 | [30] |
| *Band1* | D (Region 1) | Band1-F11<br>Band1-R6 | – | F: CGGGAGGTGGTGTACTTTCT<br>R: ACGGCTGACTGTGTGTTTGA | 60 | [70] |

**Table S4b.** Plastid microsatellite (cpSSR) primer pairs, primer sequences and annealing temperature (T°m).

| DNA marker | Location | Repeat | Sequence (5'→ 3') | T°m | References |
|---|---|---|---|---|---|
| STCP1 | *trnH/ psbA* | $A_{11}$ | F: TGCAAGCAAATACCCTCTCT<br>R: CAAAAAGGTGCTATTGCTCCT | 61→56 | [36] |
| STCP2 | *rps16/ trnQ* | $T_{13}$ | F: AGTTCCCCTCTCGACATCATTA<br>R: CCCCATTCTTTGGATTTTATCA | 61→56 | [36] |
| STCP3 | *psbK/ psbI* | $T_{10}$ | F: TAGGATATGCGATGGATGAT<br>R: CGAAGAGTTTGAGAGTAAGCA | 61→56 | [36] |
| STCP4 | *trnE/ trnT* | $T_{14}$ | F: TTCTAGCCGAGGGATCTTT<br>R: GAATTGTTTCAAGACCGACTC | 61→56 | [36] |
| STCP5 | *petL/ petG* | $T_9..A_{10}$ | F: TGTCAATTCTTGTTCATTGTC<br>R: TACGAATAATCCAGCCAAAG | 58→53 | [36] |
| STCP6 | *rpl20/ rps12* | $T_{11}$ | F: TACACCAACGGAAACCATAAA<br>R: GGATAGGATGGAA-<br>GAAGGAACT | 63→58 | [36] |
| STCP7 | *clpP/ psbB* | $T_{10}$ | F: ACTTTGAGGTGGAAACGTAA<br>R: TGAGTTTATTCATTCTGTCTTTC | 60→55 | [36] |
| STCP9 | *rps19/ rpl2* | $T_8..T_9..T_{10}$ | F: TTTTCTGCTTTTGTGTTAAG<br>R: ATAGTGATAATTTGATTCTTCG | 58→53 | [36] |
| STCP10 | *ndhF/ rpl32* | $A_{10}$ | F: AAAGATACCAGTCAATAGAGAA<br>R: CTAATAGTGTCCTCCTCAAATA | 61→56 | [36] |
| STCP11 | *ndhA intron* | $A_{10}$ | F: ATTGAATTATAGGAAAGAAAGA<br>R: AGGAGAATAGGAATGAAATAA | 57→52 | [36] |
| STCP12 | *rpl2/ trnH* | $A_{10}..A_9..A_8$ | F: CGTCGCCGTAGTAAATAGGA<br>R: CGTTCGCCCATAATTCATAA | 58→53 | [36] |
| STCP13 | *rpl2/ trnH* | $A_{10}..A_9$ | F: GCCGTAGTAAATAGGAGAGAAA<br>R: AATAAATGATTGGCTACAAAGG | 60→55 | [36] |
| NTCP6 | *rps16/ trnQ* | $A_{10}$ | F: GATTCTTTCGTATCTCGATTC<br>R: GGTTCGAATCCTTCCGTC | 63→58 | [63] |
| NTCP12 | *rps2/ rpoC2* | $T_8.A_{13}$ | F: CCTCCATCATCTCTTCCAA<br>R: ATTTATTTCAGTTCAGGGTTCC | 63→58 | [63] |
| NTCP14 | *psbM/ trnD* | $T_9$ | F: AATCCGTAGCCAGAAAAATAAA<br>R: CCGATGCATGTAATGGAATC | 63→58 | [63] |

**Table S4c.** Markers and primers of the *R1* and *R3a* genes from *S. demissum* conferring race-specific resistance to late blight and of the $Ry_{sto}$ gene from *S. stoloniferum* responsible for extreme resistance to PVY.

| Target *R* gene | Chro-mosome | DNA marker | Primer sequence (5'→ 3') | T°m | Product size (bp) | References |
|---|---|---|---|---|---|---|
| *R 1* | V | R1 | F: CACTCGTGACATATCCTCACTA<br>R: CAACCCTGGCATGCCACG | 65→60 | 1400 | [64,65] |
| *R3a* | XI | RT-R3a | F: ATCGTTGTCATGCTATGAGATTGTT<br>R: CTTCAAGGTAGTGGGCAGTATGCTT | 61→56 | 982 | [66] |
| $Ry_{sto}$ | XII | YES3-3B | F: TAACTCAAGCGGAATAACCC<br>R: CATGAGATTGCCTTTGGTTA | 57→52 | 284 | [68] |
